# Current Evidence and Practice Guidelines of Systemic Complications of 2022 Mpox Outbreak: A Scoping Review

**DOI:** 10.7759/cureus.45754

**Published:** 2023-09-22

**Authors:** Kunal Ajmera, Harshal Shah, Prabal Chourasia, Satyakant Chitturi

**Affiliations:** 1 Hospital Medicine, Sentara Northern Virginia Medical Center, Woodbridge, USA; 2 Internal Medicine, Doctors Hospital of Augusta, Augusta, USA; 3 Hospital Medicine, Mary Washington Hospital, Fredericksburg , USA; 4 Hospital Medicine, Doctors Hospital of Manteca, Manteca, USA; 5 Family Medicine, California Health Care Facility, Stockton, USA

**Keywords:** monkeypox treatment, monkeypox ent complications, monkeypox ocular complications, monkeypox neurological complications, monkeypox gastrointestinal complications, monkeypox cardiovascular complications, monkeypox prevention, systemic complications monkeypox, monkeypox outbreak, monkeypox

## Abstract

In May of 2022, the World Health Organization declared a worldwide Mpox virus (MPXV) outbreak. Due to the widespread implementation of vaccination protocols and heightened awareness among the general population, there has been a notable decline in the incidence of Mpox (formerly known as Monkeypox) cases since March 2023. Nevertheless, it is crucial to remember that Mpox has the potential to impact multiple physiological systems in humans, encompassing the cardiovascular, gastrointestinal, ear/nose/throat, and ocular systems. The mortality rate of the Mpox disease is comparatively lower than that of smallpox. However, it is essential to note that this disease can still lead to significant systemic consequences. The specific pathophysiological mechanisms by which the virus affects various physiological systems are now being investigated. Direct inoculation through mucosal damage or intranasal exposure, direct viral toxicity, and lymphatic transmission via the seminal fluid are all viable hypotheses. The prompt recognition of such complications is crucial to decrease morbidity and mortality.

## Introduction and background

On May 13, 2022, the World Health Organization (WHO) received the initial report on Mpox (formerly known as Monkeypox) caused by the Mpox virus (MPXV), which subsequently led to the identification of a global outbreak. MPXV is a zoonotic double-stranded DNA virus of the family Poxviridae [1]. As of August 19, 2023, the WHO has recorded 89,391 laboratory-confirmed cases and 662 probable cases, with 153 reported deaths [2]. It is worth mentioning that a significant number of these cases can be traced back to nations that had not previously recorded transmission of Mpox. This marks the initial occurrence of Mpox cases and continuous transmission networks in countries lacking immediate or direct epidemiological connections to the West or Central African areas.

The incidence of Mpox cases has experienced a notable decrease in recent months, primarily attributed to the widespread administration of the JYNNEOS vaccine and heightened public awareness regarding the disease. The weekly average of reported cases has decreased to 102 since March 2023, in stark contrast to the peak of 7,576 cases observed during the week of August 8, 2022. The American Region exhibited the highest level of impact, with a total of 490 reported cases and 31 fatalities. Subsequently, the Western Pacific Region documented 364 instances, although no fatalities were reported within this region. During this specified time frame, the African Region documented a total of 265 cases and three fatalities [2]. The fatality rate of this condition ranges from 1% to 10%, making it comparatively less lethal than smallpox. From 1,814 cases in July 2022 to 30,671 cases as of August 2023, the number of confirmed Mpox cases in the US has increased, with California, New York, and Texas leading the way [3,4]. Most cases involve African Americans (32.89%) and the adult male population with the predominant sexual (men having sex with men) transmission route [4,5]. 

Our previously published articles have discussed detailed information on the MPXV, historical outbreaks, pathophysiology, signs/symptoms, prevention, and management of the disease [3,6]. This all-encompassing review was written with the objective of illuminating the various facets of the current Mpox outbreak and its systemic repercussions.

## Review

Mpox is classified phylogenetically into three distinct clades: Clade I and Clade IIa/IIb.

Clade I

This clade is predominantly observed in the Congo Basin region. It is worth noting that among Mpox strains, those in Clade I exhibit the highest virulence and higher severity [7].

Clade IIa/IIb

This clade is primarily found in West Africa and displays lower virulence. Clade IIb is identified as the primary causative agent of the current global outbreak in 2022.

In terms of clinical presentation, Mpox often manifests with prominent dermatological symptoms. Patients typically experience an exanthematous rash, which undergoes distinct stages of development, including macular, papular, and vesicular stages, before eventually drying out after approximately three weeks, leading to crusting. Moreover, some Mpox patients may develop concomitant symptoms such as lymphadenopathy (enlarged lymph nodes) and fever. These additional symptoms, alongside the dermatological manifestations, contribute to the overall clinical picture of Mpox [5,6].

MPXV is commonly regarded as a disease with milder exanthema and lower mortality than smallpox; However, it is crucial to recognize that beneath this seemingly milder exterior, MPXV can lead to critical systemic complications that can be life-threatening for individuals. While systemic complications can occur in both immunocompetent and immunocompromised hosts, it is common to observe a higher incidence rate in individuals with compromised immune systems. The virus does this by invading endothelial cells and subsequently disseminating throughout the body [8]. Notable complications include secondary bacterial infections, myocarditis, corneal infections, and encephalitis. Timely identification of the condition is vital to minimize total morbidity and mortality.

Gastrointestinal (GI) complications of Mpox infection

GI symptoms primarily impact the pediatric population, and most patients recover well [9]. The most frequent GI symptoms are anorexia, vomiting, nausea, abdominal pain, and diarrhea. The individuals infected with Mpox belonging to clade I or IIa have a significantly greater likelihood of experiencing GI symptoms than those infected with clade IIb [10]. African studies (predominantly clade I) report a higher prevalence of nausea/vomiting and abdominal pain than non-African studies (predominantly clade II). In comparison, diarrhea has been reported equally by both African and non-African studies. The current clade IIb outbreak does not present complications such as oral ulcers and dysphagia typically associated with clade I infection [10,11]. Conversely, symptoms such as proctitis presenting as rectal/anal pain and rectal bleeding are unique to the 2022 outbreak and have not previously been associated with Mpox infection [10-12]. In many cases of Mpox infection, anal pain can be the primary symptom observed at the time of presentation. This symptom may precede other well-known symptoms of the disease, like rash, lymphadenopathy, or fever, and can potentially lead to complications such as proctocolitis, perianal abscess, or rectal perforation [13,14]. Most cases of proctitis are seen in young men (24-53 years) self-identifying as men having sex with men (MSM) and having HIV status positive or negative while on pre-exposure prophylaxis therapy [12]. One plausible explanation posits that proctitis occurs due to direct inoculation of the MPV to the trauma to anorectal mucosa during receptive anal sex or through the seminal fluid via the lymphatic route. The data reveal the isolation of replicable components of MPX virus DNA [15], as well as MPX virus, in the seminal fluid of infected patients [16-18] while ruling out cross-contamination by the absence of virus DNA or virus detection in urine and blood samples hints at a possible genital reservoir for the virus. The virus can disseminate seminal cells or gonads via passive diffusion from blood, urine, or genital lesions or via local genital replication. Overall, symptoms are modest and self-resolving, necessitating no therapy. However, hospitalization and treatment with intravenous fluids to maintain hydration for diarrhea, antibiotics for superimposed bacterial infections, and, in rare cases, surgery for life-threatening complications such as rectal perforation or perianal abscess may be required at times. 

Neurological complications of Mpox infection

Neurological symptoms typically manifest after the prodromal symptoms of vesiculopustular rash have appeared. Frequently observed neurological symptoms include headaches (most common -50 % prevalence), followed by myalgia, anorexia, and fatigue [19,20]. Photophobia, numbness, extremity weakness, and bowel/bladder incontinence can also be seen, rapidly advancing to more severe manifestations in rare instances, such as confusion, disorientation, seizures, agitation, and encephalitis [21]. Neurological complications occur mostly in unvaccinated MSM patients [20], although the available evidence remains limited due to the scarcity of cases. Timely diagnosis and treatment are crucial to prevent the escalation of these neurological complications and ensure better patient outcomes [21]. While the specific transmission mechanisms of MPXV remain unknown, research utilizing animal models has shown the presence of MPXV in brain tissue. The virus may enter the brain via intranasal inoculation through the olfactory epithelium pathway or by infecting circulating leukocytes such as macrophages and monocytes [22,23]. One of the notable observations in the cerebrospinal fluid (CSF) analysis would be lymphocytic pleocytosis, elevated CSF protein, and negative CSF MPXV PCR and/or serology. The current absence of reliable sensitivity and specificity data for serological tests makes it challenging to definitively rule out the presence of MPXV in the CSF. However, the detection of MPXV IgM in the CSF would indicate the presence of viral antigens within the central nervous system (CNS). MRI commonly shows multifocal, bilateral, ill-defined T2/FLAIR hyperintensities in specific brain regions (such as basal ganglia, cerebellum, brainstem, and white matter) with or without perilesional edema and enhancement [24-26]. Clinicians should be mindful of the possibility of CNS problems in immunocompetent unvaccinated adults infected with MPXV as timely initiation of Tecovirimat treatment with or without corticosteroids, IV immunoglobulins or plasmapheresis would be crucial in reducing morbidity and mortality.

Cardiovascular complications of Mpox infection

Cardiovascular complications typically follow a self-limiting course; however, in rare instances, they can lead to life-threatening arrhythmias, cardiac fibrosis, dilated cardiomyopathy, or cardiogenic shock, significantly impacting both morbidity and mortality [27]. MPXV-induced myocarditis, pericarditis, or myopericarditis exhibits symptoms akin to those caused by other viral etiologies, with commonly observed presenting symptoms including pleuritic chest pain, exercise intolerance, dyspnea, palpitations, and fever, necessitating a thorough cardiovascular evaluation. Common ECG findings vary, with the most commonly seen changes being ST-segment elevation, T-wave inversion, and sinus tachycardia. Cardiac biomarkers (Troponin) and C-reactive protein are often normal or elevated [28-30]. Occasionally echocardiogram will show global ventricular dysfunction with reduced ejection fraction [28,30]. Although gold standard, endomyocardial biopsy is rarely performed due to its invasive nature. The exact pathophysiology is currently the subject of research; however, direct viral damage to the myocardium and lymphocytic infiltration of the myocardium leading to myocardial edema, inflammation, and later necrosis have been proven with other viral etiologies in the past [27]. The publication of a case report connecting increased creatine phosphokinase levels to a Mpox infection is an interesting finding, and it could have significant implications for understanding the cardiovascular effects of this infection on the human body [31]. Management would include requiring no treatment and observation at home to cardiopulmonary resuscitation and ICU admission for life-threatening arrhythmias. Complications such as myocarditis or pericarditis sometimes require treatment with beta-blockers, nonsteroidal anti-inflammatory drugs (NSAIDs), and colchicine. 

Ophthalmic and ENT complications of Mpox infection

Ophthalmic complications from Mpox infection are rare but sight-threatening. They are more prevalent in unvaccinated and immunocompromized populations than in vaccinated, immunocompetent individuals. Clinicians should pay close attention to any ocular complaints in confirmed Mpox cases. Conjunctivitis (unilateral or bilateral), eyelid edema, focal conjunctival lesions/ulceration, Corneal ulcerations, keratitis, and loss of vision are commonly reported ophthalmic complications [32,33]. Decreased visual acuity, ocular pain, discharge, frontal headache, pre-auricular lymphadenopathy, foreign body sensation, and photophobia are commonly associated symptoms. Preceding orbital and peri-ocular macula-papular rash is, in fact, only seen in up to 25% of the cases [32]. The pathophysiology includes autoinoculation of the virus in a patient infected with Mpox by rubbing the eyes or use of contact lenses [34]. 

**Figure 1:**
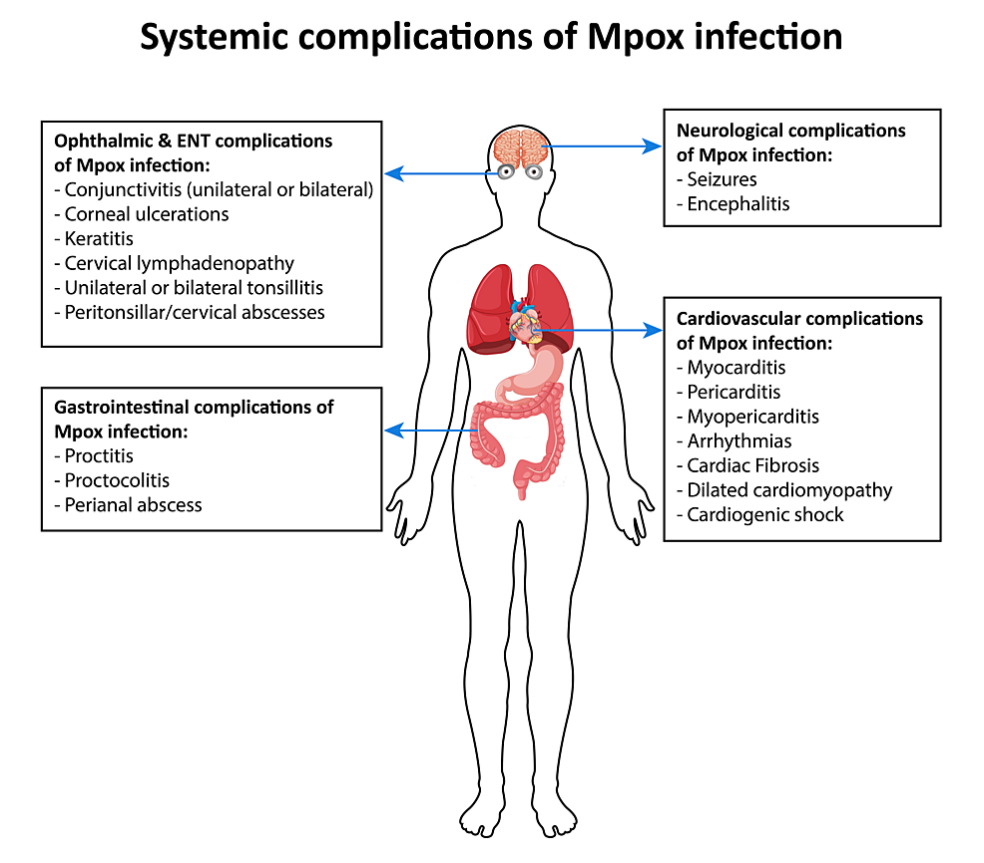
Systemic Complications of Mpox Infection. This figure is an original composition, not subject to copyright infringement.

Predominant otorhinolaryngological symptoms are headaches (most common in non-endemic areas), sore throat, odynophagia, otalgia, hearing loss, dysphagia, dyspnea, and cervical lymphadenopathy. ENT complications are not as common in the current outbreak compared to prior outbreaks and are commonly seen in the MSM group. Commonly reported complications include unilateral or bilateral tonsillitis, uvular edema, aphthous ulcer of the soft palate, ulcers in the oral cavity, and petechial edema on the hard palate [35,36]. In rare cases, the patient can develop peritonsillar/cervical abscesses and phlegmonous changes in the bilateral para-pharyngeal walls extending into the retropharyngeal space [35]. Most instances typically necessitate only outpatient care involving NSAIDs or a brief corticosteroid regimen. Hospitalization is seldom needed, primarily reserved for administering intravenous antibiotics and performing abscess drainage. Possible pathogenesis involves transmission via respiratory droplets or direct virus inoculation during high-risk sexual practice, resulting in oropharyngeal mucosal damage. Figure 1 depicts a graphical presentation of systemic complications of Mpox infection. 

Primary prevention

To prevent new cases of Mpox and future outbreaks, public health agencies and their partners must continue to improve vaccine equity and coverage among those at risk, particularly among the disproportionately represented communities of Black and Hispanic or Latino men. As per CDC data, only 23% of the at-risk population (or 1.2 million doses) has been fully vaccinated nationally as of January 2023, with the District of Columbia leading the way (97%), followed by New York City (90%) and California (66%). Some of the factors responsible for coverage heterogeneity could be lower vaccine accessibility and understanding, fewer vaccine providers, lower vaccine confidence and popularity, and stigma reservations [37].

JYNNEOS is a two-dose vaccine designed to protect against Mpox and smallpox. For optimal protection against Mpox, individuals should receive both vaccine doses. The second dose should be administered four weeks following the first. Mpox vaccination is currently recommended by the CDC for individuals at high risk of Mpox exposure, such as gay, bisexual, and other men who have sex with men (MSM) with multiple intimate partners, MSM with HIV, those with known or suspected Mpox exposure, women who have a male sexual partner who has the above risk factors, individuals with HIV or other causes of immune suppression and have had recent or anticipate future risk of Mpox exposure from any of the above scenarios, and laboratory and health care workers working with orthopoxviruses. The only contraindication is having a history of severe allergic reactions (such as anaphylaxis) to previous JYNNEOS dose. Pain, redness, and itching at the injection site are the most typical side effects of JYNNEOS immunization; however, not everyone who receives the vaccine experiences them [37].

Limitations

This review article exhibits a number of constraints. Firstly, it reflects the present state of the literature, which is subject to change as new research emerges, potentially necessitating updates to the findings presented herein. Secondly, the quality and quantity of available data serve as limitations. In some instances, the evidential support for certain topics was reliant on case reports, which inherently curtails the robustness of the evidence.

## Conclusions

The resurgence and broadening of the geographic distribution of zoonotic disease outbreaks like the ongoing Mpox outbreak are of paramount concern for public health, especially in light of the increasingly linked nature of the global ecosystem. As our world becomes more interconnected, diseases like Mpox can rapidly spread across borders, posing a substantial threat to global health security. It is vital to acknowledge that Mpox infection can manifest in various clinical symptoms, and these symptoms may vary across different clinical specialties. This highlights the complexity of Mpox and the importance of a multidisciplinary approach to understanding and managing it.

The specific pathophysiology of virus transmission is currently being researched. However, evidence points towards direct inoculation of the MPXV to the anorectal mucosa during receptive anal sex or through seminal fluid via the lymphatic route, as evidenced by the isolation of replicable components of MPX virus DNA as well as MPXV, in the seminal fluid of infected patients. The virus can then replicate locally in seminal gonads and disseminate to blood, urine, or via local genital replication. 

To prevent new cases of Mpox and future outbreaks, we must ensure that everyone at risk of Mpox has access to and is vaccinated against the virus. Healthcare professionals from various specialties must collaborate closely to identify and address the diverse range of symptoms associated with Mpox. Timely recognition of the systemic complications associated with Mpox is critical for minimizing its impact on individual health and public well-being while also preventing complications related to delayed care. Additionally, early detection is key in controlling the spread of the disease, serving as an essential component of comprehensive disease management strategies.

In essence, as we confront the challenges posed by zoonotic diseases like Mpox, interdisciplinary collaboration and a heightened awareness of the global nature of these threats are essential for safeguarding public health on a global scale.

## References

[1] Ulaeto D, Agafonov A, Burchfield J (2023). New nomenclature for mpox (monkeypox) and monkeypox virus clades. Lancet Infect Dis.

[2] (2023). 2022-23 Mpox (Monkeypox) Outbreak: Global Trends, World Health Organization.. on 20 June.

[3] Ajmera KM, Goyal L, Pandit T, Pandit R (2022). Monkeypox - an emerging pandemic. IDCases.

[4] (2023). 2022 U.S. Map & Case Count. https://www.cdc.gov/poxvirus/mpox/response/2022/us-map.html.

[5] Bragazzi NL, Kong JD, Mahroum N, Tsigalou C, Khamisy-Farah R, Converti M, Wu J (2023). Epidemiological trends and clinical features of the ongoing monkeypox epidemic: a preliminary pooled data analysis and literature review. J Med Virol.

[6] Goyal L, Ajmera K, Pandit R, Pandit T (2022). Prevention and treatment of monkeypox: a step-by-step guide for healthcare professionals and general population. Cureus.

[7] Kaler J, Hussain A, Flores G, Kheiri S, Desrosiers D (2022). Monkeypox: a comprehensive review of transmission, pathogenesis, and manifestation. Cureus.

[8] Aldhaeefi M, Rungkitwattanakul D, Unonu J, Franklin CJ, Lyons J, Hager K, Daftary MN (2023). The 2022 human monkeypox outbreak: clinical review and management guidance. Am J Health Syst Pharm.

[9] Pittman PR, Martin JW, Kingebeni PM (2022). Clinical characterization of human monkeypox infections in the Democratic Republic of the Congo (PREPRINT). medRxiv.

[10] Simadibrata DM, Lesmana E, Pratama MI, Annisa NG, Thenedi K, Simadibrata M (2023). Gastrointestinal symptoms of monkeypox infection: a systematic review and meta-analysis. J Med Virol.

[11] Huhn GD, Bauer AM, Yorita K (2005). Clinical characteristics of human monkeypox, and risk factors for severe disease. Clin Infect Dis.

[12] Yakubovsky M, Shasha D, Reich S (2023). Mpox presenting as proctitis in men who have sex with men. Clin Infect Dis.

[13] Patel A, Bilinska J, Tam JC (2022). Clinical features and novel presentations of human monkeypox in a central London centre during the 2022 outbreak: descriptive case series. BMJ.

[14] Sigle GW, Kim R (2015). Sexually transmitted proctitis. Clin Colon Rectal Surg.

[15] Lapa D, Carletti F, Mazzotta V (2022). Monkeypox virus isolation from a semen sample collected in the early phase of infection in a patient with prolonged seminal viral shedding. Lancet Infect Dis.

[16] Thornhill JP, Barkati S, Walmsley S (2022). Monkeypox virus infection in humans across 16 countries - April-June 2022. N Engl J Med.

[17] Raccagni AR, Candela C, Mileto D (2022). Monkeypox infection among men who have sex with men: PCR testing on seminal fluids. J Infect.

[18] Barboza JJ, León-Figueroa DA, Saldaña-Cumpa HM (2023). Virus identification for monkeypox in human seminal fluid samples: a systematic review. Trop Med Infect Dis.

[19] Badenoch JB, Conti I, Rengasamy ER (2022). Neurological and psychiatric presentations associated with human monkeypox virus infection: a systematic review and meta-analysis. EClinicalMedicine.

[20] Anand A, Das AK, Bhardwaj S, Singh SK (2023). A brief review of the monkeypox virus and emerging concerns for neuroinvasiveness. Surg Neurol Int.

[21] Money KM, Barnett TA, Rapaka S (2023). Monkeypox-associated central nervous system disease: a case series and review. Ann Neurol.

[22] Sepehrinezhad A, Ashayeri Ahmadabad R, Sahab-Negah S (2023). Monkeypox virus from neurological complications to neuroinvasive properties: current status and future perspectives. J Neurol.

[23] Song H, Janosko K, Johnson RF (2013). Poxvirus antigen staining of immune cells as a biomarker to predict disease outcome in monkeypox and cowpox virus infection in non-human primates. PLoS One.

[24] Pohl D, Alper G, Van Haren K, Kornberg AJ, Lucchinetti CF, Tenembaum S, Belman AL (2016). Acute disseminated encephalomyelitis: updates on an inflammatory CNS syndrome. Neurology.

[25] Young NP, Weinshenker BG, Lucchinetti CF (2008). Acute disseminated encephalomyelitis: current understanding and controversies. Semin Neurol.

[26] Sejvar JJ, Chowdary Y, Schomogyi M (2004). Human monkeypox infection: a family cluster in the midwestern United States. J Infect Dis.

[27] Jaiswal V, Sultana Q, Lahori S (2023). Monkeypox-induced myocarditis: a systematic review. Curr Probl Cardiol.

[28] Dumont M, Guilhou T, Gerin M (2023). Myocarditis in monkeypox-infected patients: a case series. Clin Microbiol Infect.

[29] Brouillard P, Valin-Thorburn A, Provost Y, Chakravarti A, Honos G, Tournoux F, Tremblay C (2022). Monkeypox associated myocarditis: a case report. IDCases.

[30] Tan DH, Jaeranny S, Li M (2022). Atypical clinical presentation of monkeypox complicated by myopericarditis. Open Forum Infect Dis.

[31] Pipitò L, Cascio A (2022). Monkeypox virus infection and creatine phosphokinase increase: a case from Italy. Travel Med Infect Dis.

[32] Ogoina D, Iroezindu M, James HI (2020). Clinical course and outcome of human monkeypox in Nigeria. Clin Infect Dis.

[33] Weppelmann TA, Wadia HP, Espana EM (2023). Large conjunctival ulceration from ocular monkeypox. Am J Ophthalmol.

[34] Cash-Goldwasser S, Labuda SM, McCormick DW (2022). Ocular monkeypox - United States, July-September 2022. MMWR Morb Mortal Wkly Rep.

[35] Alegre B, Jubés S, Arango N, Pastene D, Lehrer E, Vilaseca I (2023). Otorhinolaryngological manifestations in monkeypox. Acta Otorrinolaringol Esp (Engl Ed).

[36] Shah J, Saak TM, Desai AN (2023). Otolaryngologic manifestations among MPOX patients: a systematic review and meta-analysis. Am J Otolaryngol.

[37] (2023). JYNNEOS Vaccine Coverage by Jurisdiction. https://www.cdc.gov/poxvirus/mpox/cases-data/mpx-jynneos-vaccine-coverage.html.

